# Glutaraldehyde test for the rapid diagnosis of pulmonary and extra-pulmonary tuberculosis in an area with high tuberculosis incidence

**DOI:** 10.1590/0074-02760160541

**Published:** 2017-11

**Authors:** Ben Hadj Hassine Ahmed, Marzouk Manel, Dhaou Mohamed, Boukadida Jalel

**Affiliations:** 1 University Hospital Farhat Hached, Laboratory of Microbiology and Immunology, Sousse, Tunisia; 2 University of Monastir, Faculty of Pharmacy of Monastir, Monastir, Tunisia

**Keywords:** diagnosis, gelification time, glutaraldehyde test, light-emitting diode, tuberculosis

## Abstract

**BACKGROUND:**

Tuberculosis (TB) remains one of the leading causes of morbidity and mortality worldwide. The primary method for controlling TB is the rapid and accurate identification of infected individuals. Immune response exploitation represents one of the main methods used for early TB diagnosis; however, few studies have reported that whole blood originating from TB-infected patients gels faster in the presence of aldehyde than blood originating from healthy subjects, which is the focus of the current study.

**OBJECTIVES:**

The study objectives are to determine the diagnostic value of a glutaraldehyde test (GT) in pulmonary tuberculosis (PTB) and extra-pulmonary tuberculosis (EPTB) and to assess its performance compared with light-emitting diode fluorescence microscopy (LED-FM).

**MATERIALS AND METHODS:**

This study included 272 specimens (176 suspected PTB specimens and 96 suspected EPTB specimens). Of the 272 patients, 98 patients had TB infection confirmed by culture (64 PTB cases and 34 EPTB cases), and 174 patients had no TB infection. The gold standard technique (culture) was used as reference to verify the GT's performance.

**RESULTS:**

The GT showed a high sensitivity (96.9%) and specificity (82.1%) for PTB with a good positive predictive value (PPV = 75.6%) and negative predictive value (NPV = 97.9%). For EPTB, the GT showed a sensitivity of 91.2% and a specificity of 77.4%, with PPV = 68.9% and NPV = 94.1%. LED-FM had lower sensitivities for PTB (65.6%) and EPTB (42.1%) and an excellent specificity of 100%, with PPV = 100% and NPV = 100%.

**CONCLUSION:**

We concluded that GT is rapid, easy, simple and cost-effective and does not require qualified personnel with a specific background or sophisticated equipment like molecular biology or mycobacterium-specific genotyping techniques. These qualities make the GT attractive for use in low- and high-income countries in addition to other conventional methods, particularly culture, which continues to be the gold standard.

Tuberculosis (TB) remains one of the leading causes of morbidity and mortality worldwide (WHO 2016a). The primary method for controlling TB is the rapid and accurate identification of infected individuals. Diagnosis of TB is based on clinical symptoms, chest X-rays and tuberculin skin tests but ultimately requires the detection of the causative agent by direct microscopy examination of biological specimens and *in vitro* cultures [WHO (2016b) n.d.]. Smear examination and culture of *Mycobacterium tuberculosis* bacilli have remained the gold standard. The sensitivity of the direct smear examination of sputum, however, is low, while culture is time-consuming, labourious and not always available. Hence, a sensitive and specific diagnostic test for the rapid identification of patients with active TB would facilitate the early treatment and prevention of transmission. For the eradication of the disease, it is important to improve diagnostic techniques to detect the disease at an early stage. New, rapid diagnostic tests have thus attracted the attention of laboratories all over the world, but most of these require sophisticated laboratory equipment and skilled technicians (Alavi-Naini et al. 2009). The lack of accuracy and availability of these tests, especially in developing countries, provides good reason to try other reliable and easy approaches.

Therefore, the exploitation of the immune response represents one of the primary methods for early TB diagnosis. Since 1988, Larsson (1988) reported that whole blood originating from TB-infected patients gels faster in the presence of aldehyde than blood originating from healthy subjects. This feature is related to an increase in fibrinogen and polyclonal gammaglobulins in the blood; aldehydes condense quickly with these two more basic proteins.

The objectives of the current study were to (i) evaluate the usefulness of a rapid glutaraldehyde test (GT) (GA test, ANDA-tb GA test case finding; ANDA biological, Strasbourg, France) for patients with pulmonary (PTB), (ii) evaluate its performance in cases of extra-pulmonary TB (EPTB) (currently, to the best of our knowledge, this is the first research in the world to study EPTB), and (iii) assess its performance compared with direct microscopy using LED-FM.

## MATERIALS AND METHODS

### Participants

This prospective study included samples from new patients with suspected PTB or EPTB. A case of PTB or EPTB was defined as a patient whose culture was positive for *M. tuberculosis.* We considered a positive culture for *M. tuberculosis* as a reference to verify our LED-FM and GT results. PTB and EPTB patients whose diagnoses were not confirmed by culture were excluded. Negative cases were patients with lung or tissue infections other than TB. The diagnosis of these patients was based on a negative culture for *M. tuberculosis* and clinical improvement and radiological resolution upon follow-up. Two patients were human immunodeficiency virus (HIV) seropositive.

Sputum, extra-respiratory and blood samples for the GT were preferably collected on the same day. All specimens were subjected to smear examination, and each specimen was inoculated into Lowenstein-Jensen (LJ) and Coletsos (Bio-Rad) culture media for up to eight weeks. Positive growth was confirmed by Ziehl-Neelsen staining, and mycobacterial isolates were identified using conventional biochemical techniques David et al. (1989), 198; O'Hara et al. (2003), n.d. and confirmed by GenoType molecular kits (Hain Lifescience, Nehren, Germany).

### Test methods

The GT kit (ANDA-tb GA test case finding; ANDA biological, Strasbourg, France) was used according to the protocol recommended by the manufacturer. This test reveals evidence of an increase in fibrinogen and polyclonal gamma-globulins in the blood of the analysed subject. The test utilises glutaraldehyde, a dialdehyde subject to spontaneous polymerisation over time. Whole blood originating from TB-infected patients gels faster in the presence of aldehyde than blood originating from healthy subjects. This feature is related to an increase in fibrinogen and polyclonal gammaglobulins in the blood; aldehydes condense quickly with these two more basic proteins. Blood was collected from patients in an EDTA tube. The gelification assay was performed immediately after blood was drawn to avoid the risk of spontaneous coagulation. One millilitre of whole unclotted blood was added to a GA tube. The stopper was replaced and mixed gently to avoid spontaneous haemolysis. The tube was allowed to stand and checked for gelification every two minutes, for at least 14 min, by gently tilting the tube. Evaluation of the results was recorded following the recommendations of the manufacturer. The time taken to complete the coagulation was called the blood glutaraldehyde gelification time (BGGT). Complete gelification was attained if the blood glutaraldehyde mixture did not flow when the test tube was turned upside down. A gelification time less than 600 s (10 min) denotes a possible TB infection. A gelification time between 600 (10 min) and 720 s (12 min) denotes uncertainty, and a gelification time greater than 720 s (12 min) is negative (the concentration of fibrinogen and/or gamma globulins in the analysed blood sample is not sufficiently high to induce accelerated gelification in the presence of the dialdehyde).

### Statistical methods

Statistical analyses were carried out using the EPI7.EXE epidemiology software (CDC, Atlanta, GA, USA): specificity, sensitivity, positive predictive value (PPV) and negative predictive value (NPV) were calculated for the two diagnosis method (LED-FM and GT) results; mycobacterial culture was used as the reference standard. Confidence intervals were constructed using exact methods for proportions.

### Ethics

Investigations were made, and this study was approved by the local Medical Ethics Committee of the University Hospital Farhat Hached Sousse, Tunisia.

## RESULTS

### Participants

This study was conducted in a coastal region of east-central Tunisia using blood samples originating from patients for whom specimens were submitted routinely for mycobacterial culture from university and tertiary care centres from April 1 to November 31, 2015.

A total of 272 individuals were enrolled in the study; 181 were males, and 91 were females. The mean ages of the suspected PTB and EPTB patients were, respectively, 59.6 ± 6.1 and 26.2 ± 10.1 years. Samples originating from suspected PTB patients (n = 176) were as follows: sputum (n = 150) and samples originating from the respiratory system but not classified as sputum (n = 26); and samples originating from suspected EPTB patients (n = 96): lymph node (n = 42), pleural fluid (n = 13), urine (n = 18), cutaneous biopsy (n = 8), cerebrospinal fluid (n = 10), peritoneal fluid (n = 4) and sternal puncture (n = 1).

### Tests results

Culture results were positive for *M. tuberculosis* in 64 cases of PTB. Of these 64 confirmed cases of PTB, 42 were sputum smear-positive, and 22 were sputum smear-negative (Table I). All the sputum smear-positive PTB cases (n = 42) and 20/22 sputumnegative PTB patients had positive GT results (BGGT < 600 s) (Tables I-II). The two other negative-sputum PTB patients had negative GT results (BGGT > 720 s) (Tables I-II). Twenty of the 112 patients with a pulmonary infection other than TB had a BGGT < 615 s and were considered as false positive GT tests (Table I).

**TABLE 1:** Accuracy of fluorescence microscopy and glutaraldehyde (GA) test using a culture reference standard

All specimens N = 272												
Methods			Culture		Sensitivity (%)	95% CI	Specificity (%)	95% CI	PPV (%)	95% CI	NPV (%)	95% CI
			Positive	Negative								
Fluorescence microscopy		Positive	56	0	57.1	46.8-67	100	97.3-100	100	92-100	80.6	74.5-85.5
		Negative	42	174								
	GA test	Positive	93	0	94.2	87.9-98.1	80.5	73.6-85.9	73.2	64.5-80.5	96.8	91.7-98.7
		Negative	5	174								
Respiratory specimens, N = 176												
	Fluorescence microscopy	Positive	42	0	65.6	52.6-76.8	100	95.9-100	100	89.6-100	83.6	76-89.2
		Negative	22	112								
	GA test	Positive	62	0	96.9	88.2-99.5	82.1	73.5-88.5	75.6	64.7-84.1	97.9	91.8-99.6
		Negative	2	112								
Non respiratory specimens, N = 96												
	Fluorescence microscopy	Positive	14	0	41.2	25.1-59.2	100	92.7-100	100	73.2-100	75.6	64.7-84.1
		Negative	20	62								
	GA test	Positive	31	0	91.2	75.2-97.7	77.4	64.7-86.7	68.9	53.2-81.4	94.1	82.8-98.5
		Negative	3	62								

CI: confidence interval; NPV: negative predictive value; PPV: positive predictive value.

**TABLE II:** Blood glutaraldehyde gelification time in confirmed pulmonary tuberculosis (PTB) and extra-pulmonary TB (EPTB)

Types	Blood glutaraldehyde gelification time			
	GT (+)		Uncertain GT	GT (-)
BGGT	< 300 sec	300-600 sec	600-720 sec	> 720 sec
Confirmed PTB (N = 64)	38	24	0	2
Confirmed EPTB (N = 34)	16	15	0	3

BGGT: blood glutaraldehyde gelification time; GT: glutaraldehyde test.

The culture was positive for *M. tuberculosis* in 34 cases of EPTB. Of these 34 confirmed cases of EPTB, 14 were smear-positive, and 20 were smear-negative (Table I). Thirteen smear-positive EPTB patients and 18 smear-negative EPTB patients had positive GT results (BGGT < 600 s) (Tables I-II). Three false negative GT results were noted: one false negative GT with a positive smear was provided from a 4-month-old infant with disseminated BCG infection with severe combined immunodeficiency (SCID), and the two false negative GT tests (with negative smears) were from HIV-seropositive patients who were diagnosed with meningitis TB. Among the 62 cases with an extra-pulmonary infection other than TB, 14 had a BGGT < 615 s and were considered as false positive GT results (Tables I-II).

### Estimates

The sensitivity and specificity of GT were, respectively, 96.9% and 82.1%, with PPV = 75.6% and PNV = 97.9% for PTB (Table I). The sensitivity and specificity of GT were, respectively, 91.2% and 77.4%, with PPV = 68.9% and PNV = 94.1% for EPTB (Table I).

The sensitivity and PNV of the detection of acid-fast bacilli by LED-FM in both PTB and EPTB were lower than those from the GT (Table I). However, the micros-copy method had an excellent specificity (100%) and PPV (100%) (Table I).

## DISCUSSION

Positive TB diagnosis is based on bacteriological results. One of the key steps in this diagnosis is smear microscopy, which remains the most cost-efficient tool available to diagnose TB in resource-limited high-burden settings (Affolabi et al. 2010). Unfortunately, TB microscopy is associated with low and variable sensitivity (Bonnet et al. 2011). A few years ago, the WHO recommendations included the adoption of FM in laboratories in high-income settings to improve the efficiency of smear microscopy services (Steingart et al. 2006, Khan et al. 2007, Mase et al. 2007), n.d., WHO (2016). In 2011, the WHO recommended that conventional FM be replaced by LED-FM in all settings where conventional FM is used and that LED microscopy should be phased in as an alternative to conventional Ziehl-Neelsen microscopy n.d., WHO (2016). The LED-FM is a diagnostic tool that works well without a darkroom, which has been recently developed to improve the workflow and direct microscopy methods for detecting TB (Affolabi et al. 2010, Bonnet et al. 2011, Minion et al. 2011). Although, its specificity is excellent (100% in our recent and previous studies) (Marzouk et al. 2013), its sensitivity remains insufficient, especially for extra-pulmonary samples.

Many rapid diagnostic tests, such as mycobacteriophage-based techniques (Fu et al. 2015), tests involving the polymerase chain reaction (PCR) (Ben Kahla et al. 2011, Huang et al. 2015, Deggim-Messmer et al. 2016), and serological tests (Ben-Selma et al. 2009, 2010), have shown good sensitivity and specificity, but most of these require sophisticated laboratory equipment and skilled technicians and are not currently affordable in the peripheral areas of most developing countries (Alavi-Naini et al. 2009). There is an urgent need for a simple, inexpensive, user-friendly and reliable diagnostic test for TB, in conjunction with sputum smear microscopy and X-ray methods, in the peripheral areas of developing countries (Mathur & Sachdev 2005).

Larsson et al (Larsson 1988, Larsson et al. 1990) reported that the GT might prove to be a useful diagnostic tool for TB in developing countries. They described the GT for the diagnosis of TB, with 89% sensitivity and 95% specificity (Larsson 1988, Larsson et al. 1990). This was based on the observation that after mixing with 2.5% glutaraldehyde, the EDTA-anticoagulated blood of TB patients coagulated within 10 min. It was suggested that this simple and cheap method, together with clinical examinations, might be a useful screening tool for identifying cases of PTB in peripheral health posts in developing countries where X-ray and other facilities are lacking. Larsson et al. then suggested that the BGGT could be a rapid screening method for the diagnosis of PTB.

Since then, some studies have expressed interest in the GT in cattle TB (de Kantor et al. 1993). These authors concluded that the GT in cattle is a simple, rapid and inexpensive method that could play a complementary role to the tuberculin test for detecting tuberculous cattle, especially in endemic areas with scarce resources and facilities. This specificity decreased when the test was used for animals with evidence of diseases other than TB.

However, few studies have evaluated the GT for detecting human TB (Mathur & Sachdev 2005, Alavi-Naini et al. 2009). Mathur and Sachdev (2005) and Alavi-Naini et al. (2009) reported excellent sensitivities and specificities (85.5% and 89.1%, respectively; 96.2% and 84.1%, respectively) for BGGT in human PTB. However, the fact that no reports of its kind have been published since then indicates that others may have found the test to be unreliable. Mathur and Sachdev (2005) reported that the results of the test may be affected by room temperature and showed gross variations at different hours at different room temperatures. It was, therefore, hypothesized that the temperature and time of testing might affect the results of the GT. Their findings also suggested that in conjunction with clinical examination, the GT with a standardized procedure can be used as screening test when more expensive tests are not available.

In our study, we did not consider the temperature parameter. According to the manufacturer's recommendations, the GA tubes were kept at 4°C or frozen. When in use, the GA tubes were brought to room temperature (approximately 22°C in our laboratory) and protected from direct exposure to sunlight and heat. Mathur and Sachdev (2005) reported that when BGGT < 10 min was used to indicate a positive GT, the sensitivity of the test was 84.1%, and the specificity was 96.2% at 22°C, with PPV = 91.3% and PNV = 92.6%.

Another proposed reason is that nearly all studies conducted on diagnostics for TB have focused on tests that require expensive test kits, sophisticated equipment and skilled technicians. In this era of the patenting and commercialisation of technology, most research studies are funded with the expectation of developing patentable test kits that can be sold at high prices. Such efforts do not meet the needs of poor people in developing countries who cannot afford to pay for sophisticated technology. As a result, it is not surprising that this simple test has not attracted the attention of researchers (Mathur & Sachdev 2005).

It has been reported that the validity and reliability of the improved procedure of the GT were found to be comparable to those of mycobacteriophage-based techniques, PCR-based tests and serological tests (Mathur & Sachdev 2005). The GT is thus considered to provide a useful, affordable and feasible alternative to expensive, sophisticated laboratory tests for the diagnosis of TB.

The authors reported limitations of their studies, such as the test was not evaluated in cases of EPTB, TB in HIV positive cases or childhood TB.

To the best of our knowledge, our study is the first to evaluate the GT in cases of EPTB; its sensitivity and specificity were 91.2% and 77.4%, respectively. Although the specificity of LED-FM was 100% for EPTB in our recent and previous studies (Marzouk et al. 2013), the sensitivity of the GT was much higher (91.2% vs 41.2%). These results show that even in paucibacillary samples and in conjunction with other tests, the GT can help physicians make a decision regarding the diagnosis of EPTB. A previous study from 1995 suggested that the hyperaggregation of platelets has been reported in intestinal TB, but a role for hyperactive platelets in chronic inflammatory responses was also discussed (Sarode et al. 1995).

Therefore, the exact mechanism of the rapid gelification of blood glutaraldehyde mixtures in TB is not known, but elevated fibrinogen, decreased fibrinolysis and hyperaggregation of platelets may contribute. The short gelification time should be the result of elevated levels of both gammaglobulin and fibrinogen. Elevated plasma fibrinogens with impaired fibrinolysis have decreased antithrombin III, and reactive thrombocytosis has been found in cases of TB (Robson et al. 1996). Platelet factor-4 and soluble interleukin-2 receptor alpha (sIL-2R) concentrations are high in PTB patients, indicating platelet and T-lymphocyte activation (Büyükaíjik et al. 1998). Further in-depth studies are required regarding this information.

The two HIV-seropositive patients who were in the AIDS stage had false-negative GT results. In fact, the manufacturer noted that confusing results can occur when testing samples from the AIDS stage of HIV-infection. However, our results from the HIV-seropositive sample were too weak to draw conclusions about the reliability of the GT in these patients. To the best of our knowledge, no study has been done in this context.

The GT is rapid, easy, simple and cost-effective and does not require well-qualified personnel or sophisticated laboratory equipment, which are difficult to obtain in developing countries. These qualities make this test attractive for use in low-, middle-, and high-income countries. However, this test is of little value for finding cases of TB unless combined with clinical examinations. Therefore, this test, accompanied by clinical examinations, is primarily intended as a screening tool, and it must be used in conjunction with other conventional methods, especially culture, which continues to be the gold standard for the direct positive diagnosis of TB.

The inclusion of cultured pulmonary/extra-pulmonary specimens in an automated system such as MGIT could increase the culture positivity. This was a limitation of our study.
